# Surveillance, Prevention, Evolution and Control of Emerging Viruses: A 2022 Editorial Update

**DOI:** 10.3390/v15102098

**Published:** 2023-10-17

**Authors:** Maite Freitas Silva Vaslin, Luciana de Barros Arruda, Fabricio Souza Campos

**Affiliations:** 1 Departamento de Virologia, Instituto de Microbiologia Paulo de Góes, Universidade Federal do Rio de Janeiro, Rio de Janeiro 21941-902, RJ, Brazil; 2 Laboratório de Virologia, Departamento de Microbiologia, Imunologia e Parasitologia, Instituto de Ciências Básicas da Saúde, Universidade Federal do Rio Grande do Sul, Porto Alegre CEP 90035-003, RS, Brazil

The Special Issue “Emerging Viruses: Surveillance, Prevention, Evolution and Control” has been published annually by *Viruses*, since 2019, highlighting the increasing effort of the scientific community for the surveillance and further research of new emerging or re-emerging viruses. As organisms with the fastest evolutionary rates, viruses pose challenges for many research areas relevant to human health, agricultural practices, and the world economy. The SI 2022 reflects the insights of virologists around the globe about relevant and potentially threatening new viruses, especially those arising in the edge between rural and forest biomes. Indeed, the emergence of new virus species and/or isolates infecting wild animals from the interface between forest/rural/metropolitan areas and animals from growing farms were the most prevalent issues in this SI. New arteriviruses, hepinaviruses, coronaviruses, and astroviruses were identified in rodent, vole, and gosling species, respectively [1,2,3,4]. Also, new isolates of high pathogenic H5N1 influenza virus, epizootic hemorrhagic disease virus strain (EHDV), and Lumpy skin disease virus (LSDV) were reported in wild birds and poultry, cattle, and yaks [5,6,7].

Human–animal interface in the Amazonian metropolitan area represents a constant opportunity for the emergence of new viruses. In an effort to identify viral communities in wild animals from this region, Da Paz and collaborators [1] conducted expeditions between 2014 and 2015, in the Belém metropolitan mesoregion of Pará State, Brazil, and collected serum and tissues from 39 mammals, comprising 18 marsupials, 12 rodents, and 9 chiropterans. Metagenomic analyses from pooled tissue samples obtained from rodents, morphologically identified as *Oecomys* sp., unveiled a new arterivirus, classified in the *Variarterivirinae* clade. Using a similar approach, the same group also identified a new hepinidavirus-like in the marsupials *Marmosa demerarae,* opossums, adjacent to a rural municipality of Peixe-Boi, Pará State, Brazil [2]. *Henipavirus* is a *Paramyxoviridae* genus that comprises some emerging viruses of serious public health concern. The novel virus called the Peixe-Boi virus (PBV) corroborates the circulation of henipa-like viruses far from Africa, Asia, and Australia. It was the first description of henipa-like viruses in marsupials worldwide, indicating the importance of including these animals in surveillance studies. The vulnerability of deforested environments such as those at the study site, where wild habitats overlap with rural areas, resembles the conditions in which *Hendra henipavirus* (HeV) and *Nipah henipavirus* (NiV) outbreaks arose previously [8]. 

In another effort, using next-generation sequencing, but primer-targeted to coronavirus spike, a new *Betacoronavirus* species designed as Grimsö virus was found in bank voles (*Myodes glareolus*) in Grimsö, Sweden [3]. Bank voles are among the most common rodent species in Europe and are known reservoirs for several zoonotic pathogens, such as *Puumala orthohantavirus* and *Francisella tularensis*. The study analyzed vole communities for 3 years, and the Grimsö virus was detected during this time. The phylogenetic analysis of ORF1b, S, and N genes, as well as a partial RdRp gene, suggested a relatively broad geographic distribution of CoVs in bank voles in Europe, which is indicative of possible long-term host–virus association [3]. 

In 2016, an outbreak of a novel gout disease affecting goslings emerged in China. A novel goose astrovirus (GoAstV) was associated with the disease, and successive outbreaks resulted in serious economic loss. In the study by Fu et al. [4], GoAstV was detected in multiple tissue samples from animals with clinical signs of gout in different goose farms in southern China. The authors reported the genomes of six GoAstV strains, which could be divided into two distinct clades, GoAstV-1 and GoAstV-2. GoAstV-2 is responsible for gout outbreaks in goslings and could be classified into *Avastrovirus* 3 (AAstV-3), while GoAstV-1 belongs to *Avastrovirus* 1 (AAstV-1). 

Sghaier et al. reported the re-emergence and spread of the epizootic hemorrhagic disease virus strain 8 (EHDV-8), causing epizootic hemorrhagic disease in cattle in Tunisia in 2021 [5]. The EHDV-8 strain had not been detected since 1982. The EHDV-8 strain identified in this outbreak represents a new isolate, and it was only the second time that this strain was reported. Also in Africa, this time in Botswana, a high pathogenic avian influenza virus H5N1 emerged in 2021, infecting wild birds and poultry. After active surveillance by public departments, together with the initiation of a public awareness campaign, Letsholo et al. [6] could collect data and carcasses from infected, dying, or dead birds, and fecal material from wild birds around water bodies and farms nationwide. The authors described the clinical signs observed in different species and studied the carcasses of the affected birds for the detection of influenza A. The gull genomes of four identified virus samples from distinct locations along the country were described. High-pathogenic avian influenza (HPAI) always represents a concern in wild birds and poultry, regardless of the location from which it originates. The fast identification and control of disease outbreaks in this case helped to limit epizootic spread and deleterious effects in the wild bird population and especially in the poultry industry. The absence of densely populated poultry farms in the country helped to limit the spread of this outbreak. However, the real impact on the Okavango Delta ecosystem could be to a greater extent, and only a future census will help to define the true impact on 25 threatened species that reside at the Okavango River.

In October 2021, an outbreak of suspected Lumpy skin disease virus (LSDV) occurred on a mixed yak and cattle farms in Sichuan Province, China. The outbreak resulted in high morbidity and mortality rates in yaks. Li et al. [7] detected a novel vaccine-associated recombinant LSDV infecting yaks and cattle. The recombinant strain, named China/LSDV/SiC/2021, presents at least 18 recombination events. Virus DNA was also found in *Culex tritaeniorhynchus* Giles (Diptera: *Culicidae*), indicating that it could be a vector of the virus.

Three human-infecting viruses were discussed in this SI, including the genomic surveillance of Chikungunya virus (CHIKV) in a Brazilian state [9] and the proposal of a model for broader control of pandemic viruses, based on influenza A-H1N1 and SARS-CoV-2 data [10]. CHIKV is an arbovirus from the *Togaviridae* family. Human transmission is mediated by *Aedes* spp. The virus was introduced in Brazil in 2014; however, there is still limited information about the genomic epidemiology of CHIKV from surveillance studies. Souza et al. [9] reported the recovery of 27 newly complete genome sequences from the Tocantins State in Brazil. Their study also provided a preliminary overview of the introduction and circulation of the virus within the state, with a focus on its interplay with other Brazilian states. Notably, their research highlighted the significant role of highly populated regions like the State of Rio de Janeiro, serving as an amplifier for the CHIKV and a source of dissemination to other parts of Brazil.

In a different approach, Lim and colleagues [10] developed a modeling study considering the typical viral load reported and the relative transmission rate of influenza A-H1N1 and SARS-CoV-2. Post-pandemic economic recovery drastically depends on efficient border control for safe cross-border movement. The authors refined a previously described model and predicted the relative transmission risk (TR) posed by incoming infected travelers under given measures. They observed that viruses with high typical viral loads and low transmission risk given low viral loads, such as SARS-CoV-2, are effectively controlled with moderate-sensitivity tests (ARTs) and modest quarantine periods. In contrast, viruses with low typical viral loads and substantial transmission risk at low viral loads, such as influenza A-H1N1, require high-sensitivity tests (such as polymerase chain reaction (PCR)) and longer quarantine periods.

In an interesting paper, Juma et al. [11] reported the development of a new amplicon multiplex PCR-based method for the genome surveillance of segmented RNA viruses. The use of this alternative method can help the faster identification of virus outbreaks, leading to faster responses without the need for virus cell culture enrichment. Using 74 primer pairs matching the three genomic segments of the entire Rift Valley fever virus (RVFV) (genus *Phlebovirus*, family *Phenuiviridae*) genome, they obtained around 80–90% of the genome covering of the virus from not enriched samples. The proposed amPCR method achieves sufficient sequence coverage for genotyping and other genomic epidemiological studies such as transmission dynamics.

Finally, Keck and collaborators [12] tested and compared different kinds of biosample collection cards to assess the risks of virus transmission during transport. These types of collection cards are inexpensive and useful alternatives to transport virus samples between collection sites and the laboratories used for processing. Virus inactivation and nucleic acid stabilization were analyzed at different time points for a wide variety of viruses: bluetongue virus (BTV), family *Reoviridae*, a double-stranded RNA genome with no envelope; foot-and-mouth disease virus (FMDV), family *Picornaviridae*, a single-stranded RNA genome with positive polarity; small ruminant morbillivirus, family *Paramyxoviridae*, a single-stranded RNA genome with negative polarity and lipid envelope; and lumpy skin disease virus (LSDV), family *Poxviridae*, a double-stranded DNA genome with lipid envelope. The general conclusion was that cards are safe if samples are appropriately applied. The report also described which card showed the best nucleic acid recovery.

In summary, the manuscripts featured in this Special Issue encompass studies on a wide range of viral species, offering invaluable insights into their origin, evolution, transmission patterns, and potential outcomes. These studies underscore the significance of comprehensive research not only to enhance our understanding of these novel viruses but also to enable effective strategies for their surveillance and control.
